# Improved CRISPR/Cas9 Tools for the Rapid Metabolic Engineering of *Clostridium acetobutylicum*

**DOI:** 10.3390/ijms22073704

**Published:** 2021-04-02

**Authors:** Tom Wilding-Steele, Quentin Ramette, Paul Jacottin, Philippe Soucaille

**Affiliations:** 1 INP, TBI, INSA, UPS, Université de Toulouse, 31400 Toulouse, France; wilding-@insa-toulouse.fr (T.W.-S.); ramette@insa-toulouse.fr (Q.R.); jacottin@insa-toulouse.fr (P.J.); 2 Institut National de la Recherche Agronomique (INRA), UMR 792, 31077 Toulouse, France; 3 Centre National de la Recherche Scientifique (CNRS), UMR 5504, 31400 Toulouse, France; 4 BBSRC/EPSRC Synthetic Biology Research Centre (SBRC), School of Life Sciences, The University of Nottingham, University Park, Nottingham NG7 2RD, UK

**Keywords:** CRISPR/Cas9, *Clostridium acetobutylicum*, metabolic engineering, genetic tools

## Abstract

Clustered regularly interspaced short palindromic repeats (CRISPR)/Cas (CRISPR-associated proteins)9 tools have revolutionized biology—several highly efficient tools have been constructed that have resulted in the ability to quickly engineer model bacteria, for example, *Escherichia coli*. However, the use of CRISPR/Cas9 tools has lagged behind in non-model bacteria, hampering engineering efforts. Here, we developed improved CRISPR/Cas9 tools to enable efficient rapid metabolic engineering of the industrially relevant bacterium *Clostridium acetobutylicum*. Previous efforts to implement a CRISPR/Cas9 system in *C. acetobutylicum* have been hampered by the lack of tightly controlled inducible systems along with large plasmids resulting in low transformation efficiencies. We successfully integrated the *cas9* gene from *Streptococcus* *pyogenes* into the genome under control of the xylose inducible system from *Clostridium difficile*, which we then showed resulted in a tightly controlled system. We then optimized the length of the editing cassette, resulting in a small editing plasmid, which also contained the *upp* gene in order to rapidly lose the plasmid using the *upp*/5-fluorouracil counter-selection system. We used this system to perform individual and sequential deletions of *ldhA* and the *ptb-buk* operon.

## 1. Introduction

Multiple genetic engineering tools have previously been developed for use in *Clostridium acetobutylicum*; the first tools developed were those based on the group II intron gene inactivation system [1,2]. Several different tools have also been developed for in-frame gene deletion and insertion on the basis of allele-coupled exchange; these use either replicative or non-replicative plasmids [3,4,5,6,7]. However, they are limited by time-consuming screening steps, as in all cases the chance of finding the intended modification is at best 50%; yet, in practice, this can be significantly lower. Additionally, with the exception of Foulquier et al. [3], the deletion of multiple genes at the same time is not possible.

CRISPR (clustered regularly interspaced short palindromic repeats)/Cas (CRISPR-associated proteins) tools have recently shown great potential for the genetic engineering of multiple organisms [8]; however, CRISPR/Cas systems are currently not widely used for the engineering of C. *acetobutylicum*. Initial efforts at using the CRISPR/Cas9 system from *Streptococcus pyogenes* in *C. acetobutylicum* and other related clostridia encountered issues of toxicity when trying to constitutively express *cas9* [9]. To solve this issue, researchers have used several strategies—in most cases, inducible systems have been used to control the expression of *cas9*, however, this has had limited success as current inducible systems are not sufficiently tightly controlled resulting in low transformation efficiencies [10,11,12]. A recent paper showed that a riboswitch-based system was able to efficiently control the expression of *cas9* in several different clostridia; however, this system was not tested in *C. acetobutylicum* [13].

Cas9 nickase, in which Cas9 has been mutated so that it can only cut one strand of DNA, has been shown to be less toxic and has been used successfully in several clostridial species; however, generally lower efficiencies have been reported [14,15]. Additionally, although the CRISPR/Cas9 system from *S. pyogenes* is the most widely used, other CRISPR systems have also been used, for example, CRISPR/Cpf1. CRISPR/Cpf1 systems have been reported to be less toxic, although a tightly regulated inducible system would still be required [16,17,18,19]. Some clostridia also possess endogenous CRISPR systems and these have been successfully exploited in several clostridia [20,21,22]; however, analysis of *C. acetobutylicum’s* genome showed no putative CRISPR systems. Finally, in a recent paper, an anti-Cas9 protein was used to inhibit the activity of Cas9 in *C. acetobutylicum,* as the anhydrotetracycline inducible promoter used was unable to sufficiently control the expression of *cas9* [12].

Additionally, the size of the plasmids used is also an issue; this is as having the *cas9* gene plus the editing template generally results in large plasmids over 10 kb, resulting in low transformation efficiencies. This issue can be avoided by using a two-plasmid system, in which one plasmid contains the *cas9* gene, and another plasmid which expresses the guide RNA (gRNA) and contains the editing template [11]. However, it is sometimes advantageous to overexpress one gene (on a plasmid) and delete another gene, resulting in both all in one plasmid systems and two plasmids systems being inconvenient.

In conclusion, an ideal CRISPR/Cas9 system would have the *cas9* gene under the control of a very tightly controlled inducible promoter and allow high transformation efficiencies of the editing plasmid containing the gRNA and editing template. A Cas9 cassette was constructed consisting of the *cas9* gene from *S. pyogenes* under the control of the tightly controlled xylose-inducible promoter from *Clostridium difficile*; this cassette was successfully integrated into the genome. We secondly evaluated the expression of the guide RNA and determined the minimum length of homology arms required; this allowed us to obtain very high transformation efficiencies for our editing plasmid. We managed to achieve up to 100% editing efficiency for multiple different genes.

To speed up the editing process, we also used the *upp*/5-fluorouracil counter-selection system to quickly remove the editing plasmid. Using this method, we were able to quickly delete individually and sequentially *ldhA* and the *ptb-buk* operon; this was achieved significantly faster than using currently available techniques. Finally, we demonstrated that the Cas9 cassette can be easily removed from the genome by expressing a gRNA targeting *cas9* with the relevant homology arms, restoring the region to the wild type, creating a strain with only the desired modifications.

## 2. Results and Discussion

It is clear that an ideal CRISPR/Cas9 system would need to be under the control of a tightly controlled inducible promoter; previous studies clearly showed that current inducible systems used in C. *acetobutylicum* to control the expression of *cas9* were insufficient, with the use of either NCas9 or an anti-Cas9 protein required [10,12].

A literature search was undertaken to search for other inducible systems that have previously been shown to work in clostridia. One potential candidate was the xylose-inducible system from *C. difficile* [23]. Importantly, this system was also shown to be able to efficiently control the expression of *cas9* when used in *Clostridium beijerinckii* [24].

### 2.1. Integration of Cas9 into the Genome

**Figure 1:**
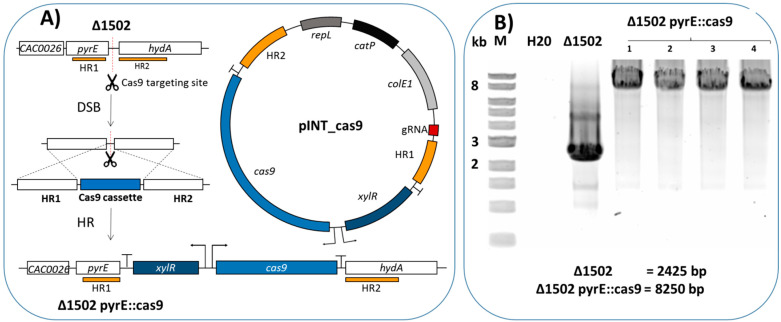
Integration of the Cas (clustered regularly interspaced short palindromic repeats (CRISPR)-associated proteins)9 cassette at the *pyrE* locus. (**A**) Schematic representation of the process of integrating the Cas9 cassette at the *pyrE* locus using the pINT_Cas9 plasmid in *Clostridium acetobutylicum*. Cas9 in combination with the guide RNA (gRNA) caused a double-strand break at the intergenic region, selecting only cells that have undergone a spontaneous homologous recombination event, resulting in the insertion of the Cas9 cassette. (**B**) PCR amplification using primers PS1 and PS2 showing the correct integration of the Cas9 cassette at the *pyrE* locus, amplification results in a 2425 bp band for the wild type, and an 8250 bp band when the Cas9 cassette was integrated. Lane M, 2-log DNA ladder (NEB); H20, water control; Δ1502, MGCΔ*cac1502* gDNA; 1–4, CAS1 (Δ*cac1502, pyrE::cas9)* clones 1–4.

We decided to integrate the Cas9 cassette into the genome in order to avoid the use of both large all-in-one plasmid systems and two-plasmid systems. The *pyrE* locus was chosen as it has previously been used for the expression of genes in *C. acetobutylicum* [7]. The wild-type *cas9* gene from *S. pyogenes* was cloned under the control of the xylose inducible system between ≈1 kb homology arms. A gRNA targeting the intergenic region between *pyrE* and *hydA* (which is not present in the plasmid) was added outside of the homology region (Figure 1A). The gRNA was placed under the control of the j23119 promoter, which has previously been used for expression of the gRNA in *C. acetobutylicum* and other clostridial species [10,13], creating plasmid pINT_Cas9 (see Figure 1A).

This plasmid pINT_Cas9 was then transformed into *C. acetobutylicum* MGCΔ*cac1502*, which is able to be transformed without any previous plasmid methylation [5]; *cas9* was then subsequently induced by plating transformants on *Clostridium* growth medium (CGM) plates containing xylose (see Section 4). Colonies growing on xylose were subsequently re-streaked twice on plates containing Reinforced Clostridial Medium (RCM) without any antibiotic in order to lose the plasmid. Once plasmid loss was confirmed by colonies being unable to grow on RCM plates containing thiamphenicol, PCR screening of four independent clones showed that the Cas9 cassette was correctly integrated, indicating 100% editing efficiency (see Figure 1B). Correct integration of the Cas9 cassette was later confirmed by DNA sequencing. The resultant strain was called CAS1 (Δ*cac1502, pyrE::Cas9*). The Cas9 cassette has subsequently been integrated into several other strains in our lab, and each time integration has been achieved with 100% efficiency.

**Figure 2:**
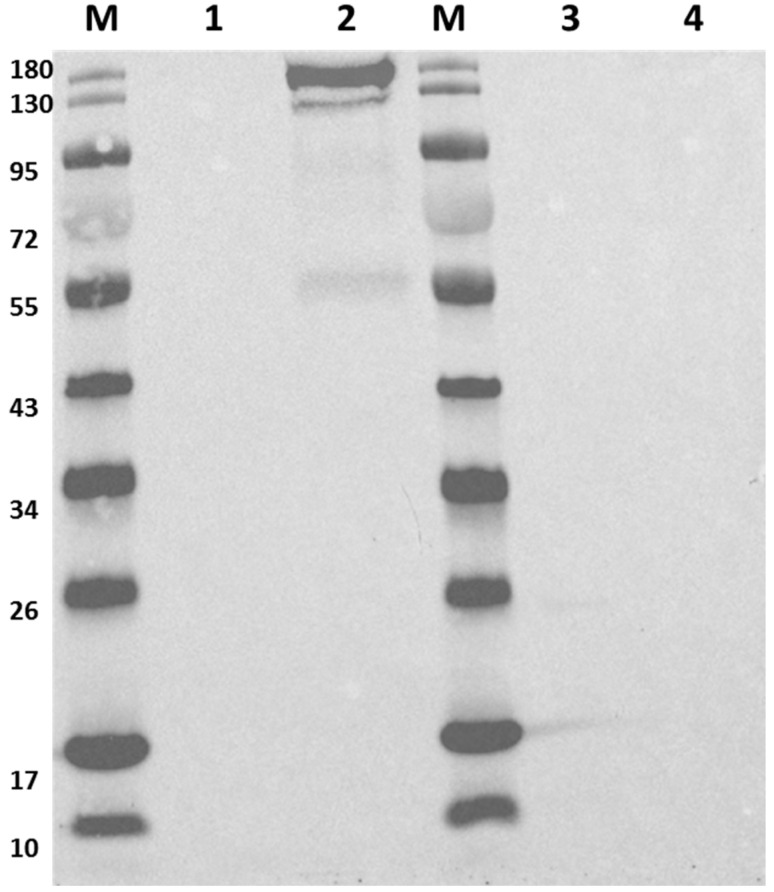
Western blot analysis of *cas9* expression in *C. acetobutylicum strains* MGCΔ*cac1502* and CAS1. The strains were grown in *Clostridium* growth medium (CGM) liquid media with either glucose or xylose as the sole carbon source; the cell pellet was then subjected to an SDS-PAGE/Western blot using an antibody raised against Cas9 (Abcam). M: marker, 1: strain CAS1 grown on glucose, 2: strain CAS1 grown on xylose, 3: strain MGCΔ*cac1502* grown on glucose, 4: strain MGCΔ*cac1502* grown on xylose. The expected molecular weight of Cas9 is 160 kDa.

Subsequently, MGCΔ*cac1502* and *CAS1* were grown in liquid CGM medium with either glucose or xylose as the sole carbon source. A Western blot using an antibody raised against the Cas9 protein was subsequently performed on crude cell extracts. For MGCΔ*cac1502*, as expected, no expression of *cas9* was detected on either glucose or xylose. On the other hand, for *CAS1,* no expression of *cas9* was detected when grown on glucose, while a high level of expression was detected when grown on xylose, implying tight control of the system (Figure 2).

### 2.2. Determination of the Efficiency of the CRISPR/Cas9 System

The *upp* gene was then selected to test the efficiency of the CRISPR/Cas9 system; several vectors were constructed with the gRNA under control of either the j23119 or the miniPthl promoter [11], with and without 1000 bp homology arms, creating pGRNAminiPthl Δ*upp*_HA1000, pGRNAminiPthlΔ*upp*, pGRNAJ23119Δ*upp*HA1000, and pGRNAJ23119Δ*upp*.

These plasmids were then transformed into CAS1, along with pCons2-1 as a control. A very high transformation efficiency was seen for all plasmids (see Table 1), implying no toxicity related to the expression of the gRNA, again indicating that *cas9* is tightly controlled. This is in contrast to the low transformation efficiencies seen using previous systems, presumably due to background expression of *cas9* [12].

Individual colonies were then grown in 1 mL of CGM until an optical density (OD) of 0.6 and plated on CGM xylose plates containing the relevant antibiotic. We achieved 100% efficiency with both the j23199 and miniPthl promoter (48 clones tested for each—see Supplementary Figures S1 and S2); however, some clones appeared on the plate containing only the gRNA (15 Colony forming units (CFU)/mL compared to 2 × 10^3^ CFU/mL with homology arms). This is likely due to escape mutations in either the gRNA or Cas9 cassette. Several clones were then re-streaked twice on RCM without antibiotics in order to lose the editing plasmid. Once plasmid loss was confirmed by colonies being unable to grow on RCM plates containing thiamphenicol, several clones were saved creating strain CAS2 (Δ*cac1502*, *pyrE::cas9*, Δ*upp*).

For clostridia, normally 1000 bp homology arms have been used; however, it is possible that shorter homology arms could be used. The main advantage of short homology arms is that it becomes economically viable to order synthetic DNA containing the gRNA and homology arms, which would significantly streamline the cloning process, as currently one step of cloning needs to be done to clone the homology arms and a second step to add the gRNA sequence. Additionally, if shorter homology arms could be used, the plasmid would be significantly smaller, which could be helpful for multiplex gene editing, as it would reduce the total size of the plasmid.

Plasmids were constructed which contained 500, 250, or 100 bp homology arms with the gRNA targeting the *upp* gene under the control of the miniPthl promoter. These plasmids were subsequently transformed into CAS1. After induction, a similar number of colonies were obtained for both 1000 bp and 500 bp homology arms (2 × 10^3^ CFU/mL); however, significantly fewer clones were observed after induction for plasmids containing 250 and 100 bp homology arms (1 × 10^2^ CFU/mL).

The 500 bp homology arms had almost the same efficiency as 1000 bp with 96% efficiency, with 45 correct clones and two clones that were a mixed population of wild type and Δ*upp* (see Figure S3). For 250 bp, 91% efficiency was achieved, with 37 correct clones, 1 clone that was a mixed population, and 3 clones that were wild type (see Figure S4). On the other hand, for 100 bp homology arms, 0% efficiency was achieved (47/47, see Figure S5). This implies that for simple deletions and insertions, 500 bp homology arms can be used (see Table 2).

### 2.3. Deletion of ldhA and the ptb-buk Operon

The efficiency of the CRISPR/Cas9 system was then tested on two other genes: *ldhA* and the *ptb-buk* operon. These genes were chosen as a clean deletion of the *ptb-buk* operon has never been performed in C. *acetobutylicum*; deletion of both *ptb-buk* and *ldhA* should result in increased solvent yields, as lactate and butyrate should not be produced as side products.

**Figure 3:**
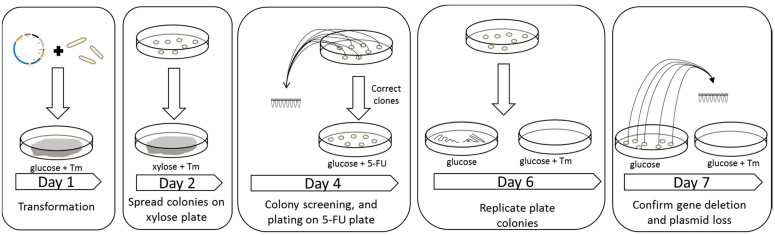
Schematic diagram of the procedure of performing genetic modifications using the CRISPR/Cas9 system in *C. acetobutylicum*. Day 1: Transformation of the strain CAS2 with a plasmid containing gRNA and homology arms. Day 2: Transformants were grown to an optical density (OD)1.0 in CGM containing glucose and then spread on a CGM plate containing xylose and relevant antibiotic. Day 4: Colonies were screened by PCR, grown until OD 1.0 in CGM containing glucose, and then spread on plates containing 5-fluorouracil (5-FU). Day 6: Colonies on 5-FU were replicated plated on plates with and without antibiotic. Day 7: Antibiotic sensitive colonies were then screened to confirm the correct genotype. The strain was then ready to be characterized and/or ready for another round of genetic modification.

Plasmids targeting *ldhA* and the *ptb-buk* operon were constructed. The gRNA targeting *ldhA* was placed under the control of the j23119 promoter while the gRNA for the *ptb-buk* operon was placed under the control of the miniPthl promoter. Additionally, to assist with the loss of the plasmid, the *upp* gene was included on the plasmid backbone; this meant that after selection on xylose the plasmid could then be easily lost by plating on plates containing 5-fluorouracil [5]. This meant it took around 7 days to delete each gene and have a strain ready for characterization or another round of genetic manipulation (see Figure 3).

The plasmids pGRNAΔ*ldhA* and pGRNAΔ*ptb-buk* were transformed into CAS2, *ldhA* was deleted with 100% editing efficiency (14/14 clones tested, see Figure S6), and the *ptb-bu*k operon was deleted with 93% efficiency (13/14 clones tested, see Figure S7). We found that 100% of colonies screened were sensitive to thiamphenicol after growth on plates containing 5-fluorouracil, creating the strains *CAS2*Δ*ldhA* and *CAS2*Δ*ptb-buk*.

**Figure 4:**
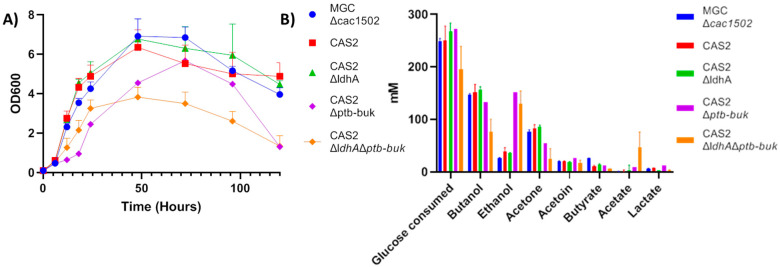
Growth curve and product analysis of recombinant strains. (**A**) Growth curve of strains MGCΔ*cac1502*, CAS2, CAS2Δ*ldhA,* *CAS2*Δ*ptb-buk*, and CAS2Δl*dhA*Δ*ptb-buk* in batch culture in synthetic medium (MS). (**B**) Solvent and acid production of MGCΔ*cac1502*, CAS2, CAS2Δ*ldhA*, *CAS2*Δ*ptb-buk*, and CAS2Δl*dhA*Δ*ptb-buk* in batch culture in MS medium.

The plasmid pGRNAΔ*ptb-buk* was then transformed into *CAS2*Δ*ldhA*, and deletion of the *ptb-buk* operon was again achieved with 93% efficiency (13/14 clones tested). We found 100% of colonies screened to be sensitive to thiamphenicol after growth on plates containing 5-fluorouracil, creating the strain *CAS2*Δ*ldhA*Δ*ptb-buk*. A growth curve and solvent analysis was then performed for strains MGCΔ*cac1502*, CAS2, CAS2Δ*ldhA,* *CAS2*Δ*ptb-buk*, and CAS2Δl*dhA*Δ*ptb-buk* (Figure 4).

Analysis of the growth curve and solvent profile of MGCΔ*cac1502* and CAS2 showed a very similar profile, indicating integrating the Cas9 cassette into the genome had no effect on growth or solvent formation again implying no or very low background expression of *cas9*.

Deletion of *ldhA* resulted in a strain with a very similar growth and solvent profile to both MGCΔ*cac1502* and CAS2 with the only difference being very low levels of lactate being produced (3 vs 7 mM) (Figure 4).

Both *CAS2*Δ*ptb-buk* and CAS2Δl*dhA*Δ*ptb-buk* grew slower compared to the control strains (Figure 4A). *CAS2*Δ*ptb-buk* produced a molar ratio of 4.5:1.5:4 of butanol, acetone, and ethanol compared to the normal ratio of 6:3:1. *CAS2*Δ*ptb-buk* also produced slightly higher levels of acetate and lactate, and as expected, also produced very low levels of butyrate.

CAS2Δl*dhA*Δ*ptb-buk* produced a molar ratio of 3:1:7 of butanol, acetone, and ethanol. The strain also produced high levels of acetate, implying the strain was not able to properly re-uptake acetate, explaining the low level of acetone produced. As expected, CAS2Δl*dhA*Δ*ptb-buk* also produced very low levels of butyrate and lactate (Figure 4B).

This is the first clean deletion of the *ptb-buk* operon with all other mutants targeting either *buk* or *ptb*. Previous mutants were created using either a disruptive non-replicative plasmid/group II intron gene inactivation system to delete either *buk* or *ptb*; these deletion systems can often disrupt the expression of neighboring genes. Previous efforts to create a clean deletion of *buk* resulted in spontaneous partial deletions of *ptb*, implying that a Δ*buk* mutant is not viable [25].

Various different phenotypes have been described in different Δ*buk*/Δ*ptb* mutants. G.N. Bennett et al. [26] inactivated *buk* using a disruptive non-replicative plasmid—the resultant strain produced higher levels of butanol and acetate compared to the wild type, with a decrease in butyrate production. In a subsequent paper, *ptb* was disrupted using a group II intron gene inactivation system—this resulted in a strain that in pH-uncontrolled fermentations produced high levels of acetate and lactate while producing no acetone or butyrate and low levels of butanol. However, in pH-controlled fermentations, the strain produced high levels of ethanol and acetone [27]. Finally, P. Soucaille et al. [25] attempted a clean deletion of *buk*; however, spontaneous mutations in *ptb* were identified. The resultant strain showed increased butanol production compared to the wild type, with no increase in ethanol or acetone production. It is possible that this difference in phenotype is due to some residual activity of *ptb* or other secondary mutations. Detailed genetic analysis and characterization of these strains would be useful to determine the reason for these differing phenotypes, however, are outside the scope of this study.

### 2.4. Removal of Cas9

Growth curve and solvent analysis showed that integration of the *cas9* gene under the control of the inducible promoter does not affect either growth or solvent production indicating that removal of the Cas9 cassette would not be routinely required. However, in certain cases it would be desirable to have a clean strain; we showed that the Cas9 cassette could easily be removed using plasmid pGRNAΔc*as9*, consisting of a gRNA targeting the Cas9 cassette along with the relevant homology arms cloned into an appropriate vector. The Cas9 cassette was successfully removed from strain CAS2 using this plasmid with 100% efficiency (14/14 clones tested, see Figure S8), restoring the *pyrE* locus to wild type.

## 3. Conclusions

In this paper, we were able to achieve high-efficiency genetic engineering of *C. acetobutylicum* by using a tightly controlled xylose inducible system. We showed that the Cas9 cassette could be quickly integrated into the genome and that this had no effect on growth or solvent formation when grown on glucose. We achieved very high editing efficiency of three different genes (*upp*, *ldhA*, and the *ptb-buk* operon) as well as the integration and subsequent removal of the Cas9 cassette. A table summarizing the editing efficiency is shown in Table 3.

This system offers significant improvements on previously developed CRISPR/Cas9 systems for *C. acetobutylicum*, mainly the use of the tightly controlled xylose-inducible promoter from *C. difficile*, resulting in no background expression of *cas9*, which was not the case for the previously described lactose and anhydrotetracycline systems. Additionally, we have optimized the size of the homology arms and shown that the editing plasmid can quickly be lost using the *upp*/5-fluorouracil system. Importantly, integration of the Cas9 cassette into the genome means only one editing plasmid is necessary, important in *C. acetobutylicum,* as only two antibiotic-resistant markers are available.

We envisage that this system will significantly decrease the time needed to genetically modify *C. acetobutylicum*, which should result in more ambitious metabolic engineering projects being attempted. With most papers only performing one or two deletions/modifications, while to our knowledge the highest number of genes modified has been eight [28].

## 4. Materials and Methods

### 4.1. Growth Conditions

*C. acetobutylicum* ATCC 824 was grown anaerobically at 37 °C in either a synthetic medium (MS), in *Clostridium* growth medium (CGM), or Reinforced Clostridial Medium (RCM), as described previously [3]. Glucose or xylose was added to the media at a final concentration of 5%. Solid media was obtained by adding 1.5% agar to the liquid media. Media was supplemented, when required, with the appropriate antibiotic at the following concentration: thiamphenicol at 15 µg/mL. 5-Fluorouracil (5-FU) was purchased from Sigma, and stock solutions were prepared in DMSO. Transformation of *C. acetobutylicum* was carried out as described previously [3].

*Escherichia coli* TOP10 was grown at 37 °C in Luria–Bertani medium, supplemented when required, with the appropriate antibiotic at the following concentration: chloramphenicol (25 µg/mL).

### 4.2. DNA Isolation and Manipulation

Total genomic DNA from *C. acetobutylicum* ATCC 824 was isolated using GenElute Bacterial Genomic DNA Kits (Sigma, Munich, Germany). Plasmid DNA was extracted from *E. coli* with the NucleoSpin Plasmid (Macherey-Nagel, Düren, Germany). DNA restriction enzymes, T4 DNA ligase, Phusion polymerase, and NEBuilder HiFi DNA Assembly were obtained from NEB (New England BioLabs, Ipswich, MA, USA). DNA fragments were purified from agarose gels with the QIAquick gel purification kit (Qiagen, Hilden, Germany). PCR was carried out using chromosomal DNA as a template using Phusion polymerase (NEB, Ipswich, MA, USA). The detailed construction of each plasmid is described in the Supplementary Materials along with a list of primers used (Supplementary Table S2), and the gRNA sequences (Table S1). A list of plasmids used is shown in Table 4.

### 4.3. Analytical Methods

Cell growth was monitored by measuring optical density at 600 nm (OD600). Solvent and acid production, as well as glucose consumption in cell-free supernatant samples, were determined on the basis of high-performance liquid chromatography (HPLC) [29] using H_2_SO_4_ at 0.5 mM as mobile phase.

### 4.4. Western Blot

To determine the expression of *cas9*, we grew the relevant strains in CGM liquid with either glucose or xylose to an OD of 1.0. A total of 10 mL of cell culture was then centrifuged at 6500 RPM (Revolutions per minute of rotor), 4 °C for 10 min. The cell pellet was then stored at –20 °C for 16 h. The cell pellet was then resuspended to an OD of 10 in Phosphate Buffered Saline (PBS). SDS-PAGE and Western blot were then performed using a Horseradish-peroxidase (HRP)-conjugated antibody raised against Cas9 from *S. pyogenes* (Abcam -ab202580).

### 4.5. Construction of Strains

After transformants were obtained on RCM media containing the relevant antibiotic, we inoculated several colonies into 1 mL of liquid CGM-glucose, and after several hours of incubation, serial dilutions were performed and 100 uL of culture was spread on solid CGM-xylose plates. After around 48 h of incubation, colonies were screened by colony PCR using relevant primers (see Table S2).

In the case of constructs in which the *upp* gene was not present on the plasmid backbone, we re-streaked individual colonies on RCM plates to obtain single colonies. Several colonies were then screened by streaking on RCM plates with and without the relevant antibiotic; colonies that grew without antibiotic but did not grow in the presence of the antibiotic were presumed to have lost the plasmid.

In the case of constructs in which the *upp* gene was present on the plasmid backbone, individual colonies were inoculated in 1 mL of CGM-glucose, and after several hours of incubation, 100 uL of culture was spread on a plate containing CGM-glucose supplemented with 2-(N-morpholino)ethanesulfonic acid _(MES) (15 g/L) and 5-FU at 0.1 mM. Colonies were then screened by streaking on RCM plates with and without the relevant antibiotic; colonies that grew without antibiotic but did not grow in the presence of the antibiotic were presumed to have lost the plasmid. The list of strains used is shown in Table 5.

## Figures and Tables

**Table 1:** Transformation efficiencies of strain CAS1 with pCons2-1, pGRNAminiPthl Δ*upp*_HA1000, pGRNAminiPthlΔ*upp,* pGRNAJ23119Δ*upp*HA1000, and pGRNAJ23119Δ*upp.* Values are expressed in number of transformants per microgram DNA. Mean values and standard deviations from three independent experiments are given. A total of 0.5 μg of DNA was used in each experiment.

Plasmid	pCons2-1	pGRNAminiPthl Δ*upp*_HA1000	pGRNAminiPthlΔ*upp*	pGRNAJ23119Δ*upp*HA1000	pGRNAJ23119Δ*upp*
CFU/μg of DNA	5.13 (±0.51) × 10^3^	5.45 (±0.67) × 10^3^	7.81 (±0.84) × 10^3^	7.53 (±0.58) × 10^3^	7.66 (±0.72) × 10^3^

**Table 2:** Comparing the editing efficiency of the CRISPR/Cas9 system targeting the *upp* gene using both the J23119 or MinPthl Promoter and either 1000, 500, 250, or 100 bp homology arms.

Promoter	J23119	MiniPthl			
Size of homology arms	1000 bp	1000 bp	500 bp	250 bp	100 bp
Editing efficiency	100%	100%	96%	91%	0%

**Table 3:** Summarizing the efficiency of genetic modifications performed using the CRISPR/Cas9 system.

Gene	Integration of Cas9 Cassette	Deletion of *upp*	Deletion of *ldhA*	Deletion of *ptb-buk* Operon	Removal of Cas9 Cassette
Efficiency	100%	100%	100%	93%	100%
size ofdeletion/insertion	5872 bp	630 bp	942 bp	2001 bp	5872 bp

**Table 4:** List of plasmids.

Plasmids	Relevant Characteristics	Source or Reference
pCons2-1	*Cm^r^, repL*	[5]
pCons::*upp*	*Cm^r^, repL, upp*	[5]
pINT_*cas9*	*Cm^r^, repL*, *pyrE*::pxyl_*cas9*	This study
pGRNAminiPthl Δ*upp*_HA1000	*Cm^r^, repL,* miniPthlgRNA*upp*, Δ*upp*	This study
pGRNAminiPthlΔ*upp*	*Cm^r^, repL*, miniPthlgRNA*upp*,	This study
pGRNAJ23119Δ*upp*HA1000	*Cm^r^, repL*, J23119PthlgRNAupp	This study
pGRNAJ23119Δ*upp*	*Cm^r^, repL*, J23119PthlgRNA*upp*, Δ*upp*	This study
pGRNAminiPthl Δupp_HA500	*Cm^r^, repL*, miniPthlgRNA*upp*, Δ*upp*	This study
pGRNAminiPthl Δ*upp*_HA250	*Cm^r^, repL*, miniPthlgRNA*upp*, Δ*upp*	This study
pGRNAminiPthl Δ*upp*_HA100	*Cm^r^, repL*, miniPthlgRNA*upp*, Δ*upp*	This study
pGRNAΔ*ldhA*	*Cm^r^, repL*, *upp*, J23119gRNA*ldhA*, Δl*dhA*	This study
pGRNAΔ*ptb-buk*	*Cm^r^, repL, upp*, miniPthlgRNA*buk*, Δ*buk-ptb*	This study
pGRNAΔ*cas9*	*Cm^r^, repL*, *upp*, J23119gRNA*cas9*, Δ*cas9*	This study

**Table 5:** List of strains.

Strain	Relevant Characteristics	Source or Reference
Bacterial strains		
*E. coli TOP10*		Invitrogen
*C. acetobutylicum*		
MGCΔ*cac1502*	Δ*CA_C1502*	[5]
CAS1	ΔCA_C1502, pyrE::pXyl_cas9	This study
CAS2	ΔCA_C1502, pyrE::pXyl_cas9, ΔCA_C2879	This study
CAS2Δ*ldhA*	ΔCA_C1502, pyrE::pxyl_cas9, ΔCA_C2879, ΔCA_C0267	This study
CAS2Δ*ptb-buk*	ΔCA_C1502, pyrE::pxyl_cas9, ΔCA_C2879, ΔCA_C3075, ΔCA_C3076	This study
CAS2Δl*dhA*Δ*ptb-buk*	ΔCA_C1502, pyrE::pxyl_cas9, ΔCA_C2879, ΔCA_C0267, ΔCA_C3075, ΔCA_C3076	This study

## Supplementary Materials

Supplementary materials can be found at https://www.mdpi.com/article/10.3390/ijms22073704/s1.
