# Supplementary material for: Improved CRISPR/Cas9 Tools for the Rapid Metabolic Engineering of Clostridium acetobutylicum
Source: Int J Mol Sci. 2021 Apr 2;22(7):3704. doi: 10.3390/ijms22073704 (PMC8037352; doi:10.3390/ijms22073704)
Supplement: Supplementary file 1 [file ijms-22-03704-s001.pdf]

Supplementary materials

Improved CRISPR/Cas9 Tools for the Rapid Metabolic Engineering of *Clostridium acetobutylicum*

Tom Wilding-Steele [1,2,3], Quentin Ramette [1,2,3], Paul Jacottin [1,2,3] and Philippe Soucaille [1,2,3,4,*]

[1] INP, LISBP, INSA, UPS, Université de Toulouse, 31400 Toulouse, France; wilding-@insa-toulouse.fr (T.W.-S.); ramette@insa-toulouse.fr (Q.R.); jacottin@insa-toulouse.fr (P.J.)
[2] Institut National de la Recherche Agronomique (INRA), UMR 792, 31077 Toulouse, France
[3] Centre National de la Recherche Scientifique (CNRS), UMR 5504, 31400 Toulouse, France
[4] BBSRC/EPSRC Synthetic Biology Research Centre (SBRC), School of Life Sciences, The University of Nottingham, University Park, Nottingham NG7 2RD, UK
* Correspondence: soucaille@insa-toulouse.fr

Cloning of constructs

A cassette consisting of a xylose inducible promoter and repressor from *C. difficile*, the wild-type *cas9* gene from *S. pyrogenes*, flanked by terminators along with homology arms, and a gRNA targeting the intergenic region under control of the j23119 promoter along with flanking SapI sites was synthesized (see sequence cas9 cassette). pCons2-1 was digested with SapI along with the cas9 cassette, followed by ligation creating pINT_Cas9. To construct pConsΔupp_HA1000, pCons2-1 [5] was digested with BamHI/EcoRI, the homology arms for *upp* were PCR amplified from strain MGC_del1502 [5] using primers PC1 and PC2. The PCR product was digested with BamH1/EcoR1 followed by ligation. The gRNA sequence consisting of the promoter (either j23119 or miniPthl), 20 bp gRNA sequence and scaffold were synthesized along with relevant cloning sites. This was then cloned into pConsΔupp_HA1000 by digesting the synthetized gRNA and pConsΔupp_HA1000 with Apa1 and BamH1 followed by ligation, creating pGRNAminiPthlΔ*upp*_HA1000 and pGRNAJ23119Δupp_HA1000.

pGRNAminiPthlΔupp_HA500, pGRNAminiPthlΔupp_HA250 and pGRNAminiPthlΔ*upp*_HA100 were cloned by amplifying the relevant homology arms using primer pairs PC3 and PC4 for 100 bp, PC5 and PC6 for 250bp and PC7 and PC8 for 500 bp. pGRNAminiPthlΔ*upp*_HA1000 and the PCR products were digested with BamHI/EcoRI followed by ligation.

pGRNA*ΔldhA*, pGRNA*Δptb-buk* and pGRNA*Δcas9* were cloned using pCons::upp [5] as a template, the gRNA was synthesized and added as described for *upp*. For pGRNA*ΔldhA* the homology arms were amplified using primers PC13 and PC14 using strain ΔCA_C 1502ΔCA_C 2879ΔCA_C 3535ΔCA_C 0267 [5] as a template, the PCR product and backbone were then digested with BamHI/EcoRI followed by ligation. For pGRNA*Δcas9* the relevant homology arms were amplified by PCR using wild-type *C. acetobutylicum* gDNA for a template using primers PC15 and PC16, the PCR product and backbone were then digested with BamHI/EcoRI followed by ligation. For pGRNAΔ*ptb-buk* the backbone was digested with BamHI/EcoRI and the relevant homology arms were than cloned using GeneArt™ Seamless Cloning and Assembly kit, using primers PC9 to PC12.

Table S1. A list of gRNA sequence used to target each gene.

Target	gRNA sequence
upp	ACTAAATGTAAAATGTTAGC
ldhA	AGTTGGAATTAACGGAGTGA
buk-ptb	TTAATCGTAGTCCACATGGG
cas9	CTCGTAGAAGGTATACACGT

Table S2. List of PCR primers used.

**Screening primers**		
PS1	pyrEscreen_Fw	TGTTGGAACAGAAATAGCTGGATGT
PS2	pyrEscreen_Rev	ACCAGAAGATAAGGATGCTCTAGTTGA
PS3	UPPscreen_Fw	CTCTATCAGGCGGACAAAAGCAAAA
PS4	UPPscreen_Rev	CTTTTGACGAAGAAGGCTTTGGAGT
PS5	ldhAsreen_fw	GGGAAAGGTTTTAAGAGCGGCG
PS6	ldhAscreen_rev	CAACAATTGTCTCCGGTTTCAAGGG
PS7	bukptbscreen_Fw	ACATGGGGCCTGACATTTCATTTTT
PS8	bukptbscreen_Rev	GGATCCTAGATGCACGTATGTTTTAGAAG
**Cloning primers**		
PC1	UPP_BAMHI_Fw	ACTAGTGGATCCATAATATGTGTAGAACATAATTTAAAGGC
PC2	UPP_EcoR1_Rev	GTAATGGAATTCTTACTTGGTTTTATAGAGATTTTAAGG
PC3	UPP_100_FW	CTATATGGATCCTAATTTCTATTATTATCAGAAGAGGCA
PC4	UPP_100_REV	ATGCATGAATTCGTAGATACATTTTTAAATTCAAAATTTCAGGG
PC5	UPP_250_FW	CTCCAGGATCCATGCAGTTTAAAAAGGGATTTAAGT
PC6	UPP_250_REV	CATCTGAATTCGAATACTCATTTGTGGAACAGGTATAGG
PC7	UPP_500_FW	TGTGCTGGATCCACTGTTGGTAAAAGTGATCTCG
PC8	UPP_500_REV	TCTGCTGAATTCATCTTCTTTTTTTGCGATTATGTAT
PC9	LH-delbukptb_fwd	CGGCCGCTCTAGAACTAGTG AACAGGACTTAAGAATATTATTCC
PC10	LH-delbukptb_rev	TGTACGACCATAAAGTCATAAATAATATAATATAACCAGTAC
PC11	RH-delbukptb_fwd	TATGACTTTATGGTCGTACACTCCCTTTTAC
PC12	RH-delbukptb_rev	CACGGATCTGGATCATTACGAAATGGAGCTGAGATTCCATG
PC13	ldh_ecor1	GTAATGGAATTCGTCGACAAAAAAGCACCGACTCG
PC14	ldh_bamh1	CTCTTTCCAAATTTTAAAGCGGATCCAGATCC
PC15	PyrE_BAMH1_Fw	GCATGGGATCCGGTGGAGAGTAATGTACTTACCTTTGGG
PC16	PyrE_Ecor1_rev	GCTGCTTTAAAAGAAAAATCCC

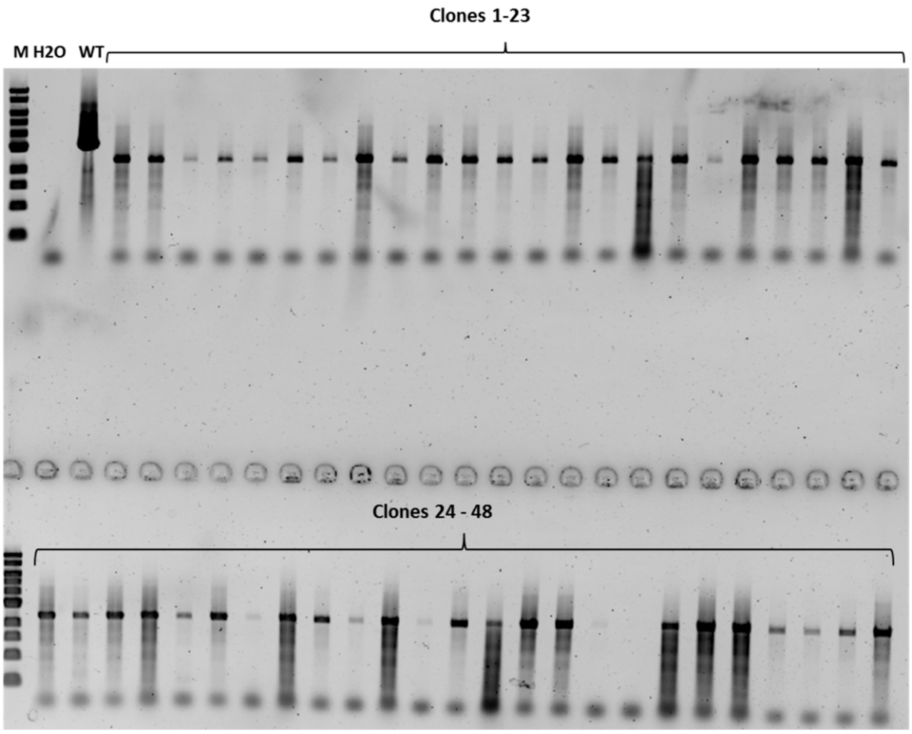

Figure S1. - Deletion of *upp* in strain *CAS1* using pGRNAminiPthlΔ*upp*_HA1000. PCR amplification using primers PS3 and PS4 showing the correct deletion of the *upp* gene. Amplification results in a 3200 bp band for the wild-type and a 2500 bp band when the *upp* gene is deleted. Lane M; 2-log DNA ladder (NEB), H20; water control, WT; MGCΔ*cac1502* gDNA, 1-48 – Clones 1 -48 showing correct deletion of *upp.*

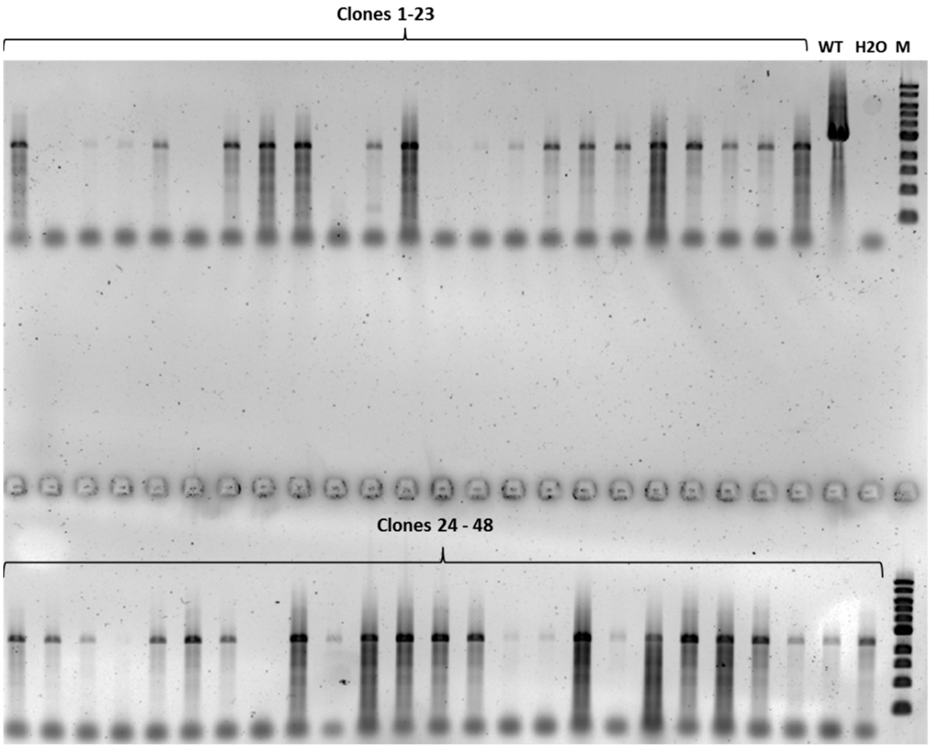

Figure S2. - Deletion of *upp* in strain *CAS1* using pGRNAJ23119Δupp_HA1000. PCR amplification using primers PS3 and PS4 showing the correct deletion of the *upp* gene. Amplification results in a 3200 bp band for the wild-type and a 2500 bp band when the *upp* gene is deleted. Lane M; 2-log DNA ladder (NEB), H20; water control, WT; MGCΔ*cac1502* gDNA, 1-48 – Clones 1 -48 showing deletion of *upp.*

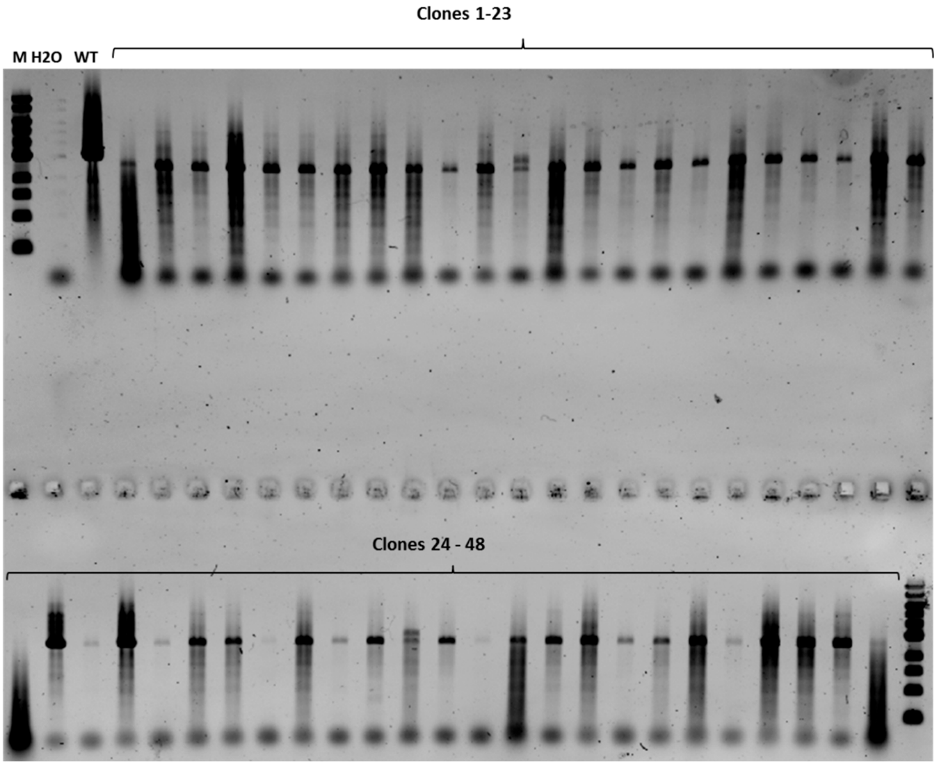

Figure S3. – Deletion of *upp* in strain *CAS1* using pGRNAminiPthlΔupp_HA500. PCR amplification using primers PS3 and PS4 showing the correct deletion of the *upp* gene. Amplification results in a 3200 bp band for the wild-type and a 2500 bp band when the *upp* gene is deleted. Lane M; 2-log DNA ladder (NEB), H20; water control, WT; MGCΔ*cac1502* gDNA, 1-48 – Clones 11 and 36 show a mixed genotype of wild-type/Δ*upp,* clone 24 was excluded as no amplification can be seen, all other clones show a Δ*upp* genotype.

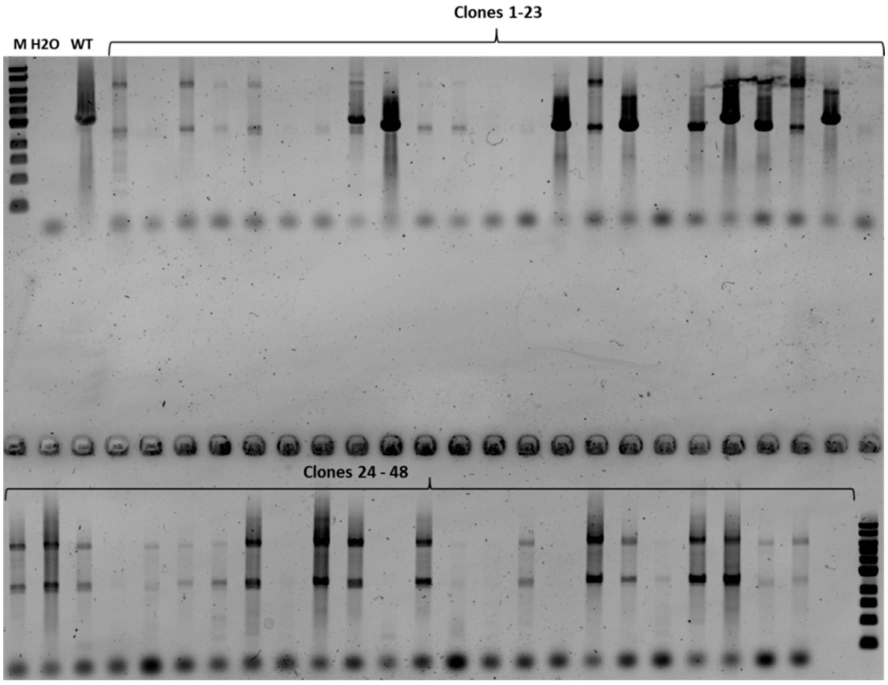

Figure S4. - Deletion of *upp* in strain *CAS1* using pGRNAminiPthlΔupp_HA250. PCR amplification using primers PS3 and PS4 showing the correct deletion of the *upp* gene. Amplification results in a 3200 bp band for the wild-type and a 2500 bp band when the *upp* gene is deleted. Lane M; 2-log DNA ladder (NEB), H20; water control, WT; MGCΔ*cac1502* gDNA, 1-48 – clone 8 showed a mixed genotype, clones 19 and 22 were wild-type, while clones 2, 6, 12, 17, 32, 35, 40 and 48 were excluded as no amplification could be seen.

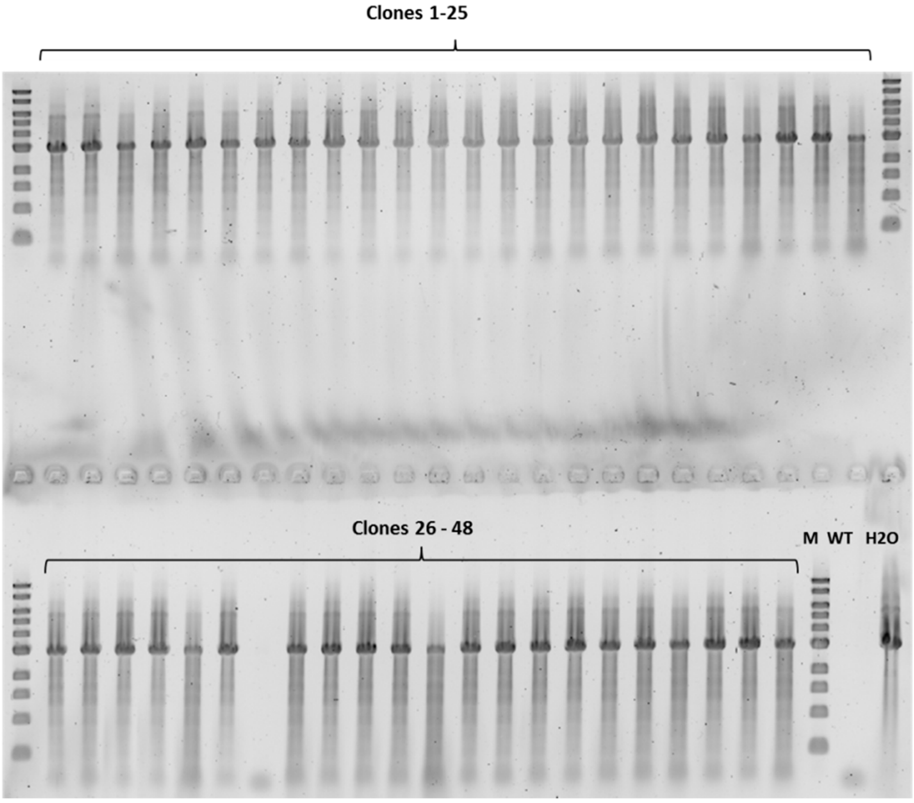

Figure S5. - Deletion of *upp* in strain *CAS1* using pGRNAminiPthlΔ*upp*_HA100. PCR amplification using primers PS3 and PS4 showing the correct deletion of the *upp* gene. Amplification results in a 3200 bp band for the wild-type and a 2500 bp band when the *upp* gene is deleted. Lane M; 2-log DNA ladder (NEB), H20; water control, WT; MGCΔ*cac1502* gDNA, 1-48 – Clone 33 was excluded as no amplification was observed. All other clones were wild-type.

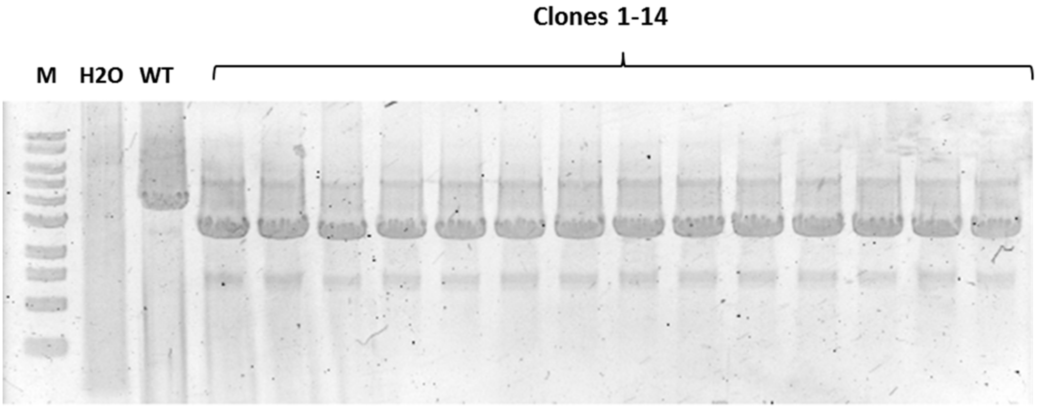

Figure S6. – Deletion of *ldhA* in strain CAS2 using pGRNA*ΔldhA.* PCR amplification using primers PS5 and PS6 showing the correct deletion of the *ldhA* gene. Amplification results in a 4000 bp band for the wild-type and a 3000 bp band when the *ldhA* gene is deleted. Lane M; 2-log DNA ladder (NEB), H20; water control, WT; MGCΔ*cac1502* gDNA, 1-14 = correct deletion of *ldhA.*

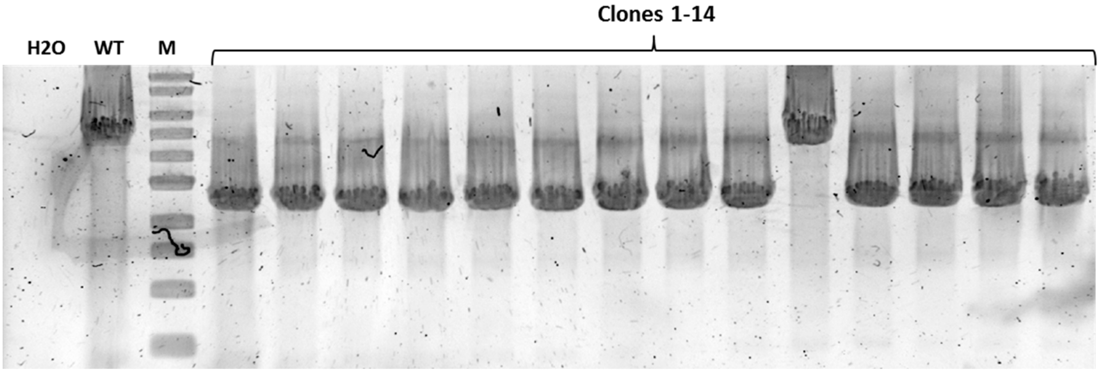

Figure S7. – Deletion of the *ptb-buk* operon in strain CAS2 using pGRNA*Δptb-buk.* PCR amplification using primers PS7 and PS8 showing the correct deletion of the *ptb-buk operon*. Amplification results in a 4800 bp band for the wild-type and a 2800 bp band when the *ptb-buk operon* is deleted. Lane M; 2-log DNA ladder (NEB), H20; water control, WT; MGCΔ*cac1502* gDNA, 1-14 = Clone *10* showed a wild-type genotype, while all other clones showed deletion of the *ptb-buk* operon.

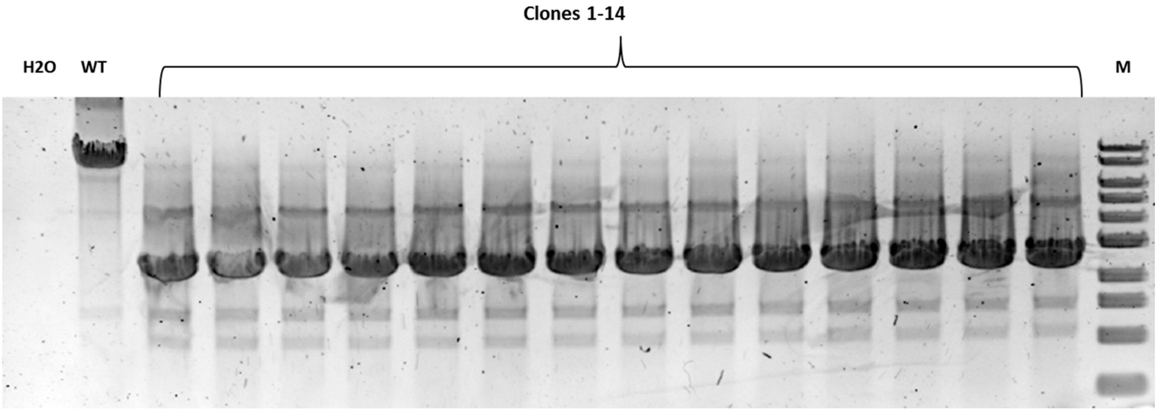

Figure S8. – Removal of *cas9* using pGRNA*Δcas.* PCR amplification using primers PS1 and PS2 showing the correct removal of the Cas9 cassette at the *pyrE* locus, amplification results in a 8250 bp band for strain CAS1 and an 2425bp band when the Cas9 cassette has been removed. Lane M; 2-log DNA ladder (NEB), H20; water control, WT; CAS1 , 1-14; Clones 1-14 where PCR amplification indicates correct removal of the Cas9 cassette. .

Sequence of Cas9 cassette

GAAGAGCGGGCCCTTGACAGCTAGCTCAGTCCTAGGTATAATACTAGTGTGCCTTAACATCTAAGTTG
GTTTTAGAGCTAGAAATAGCAAGTTAAAATAAGGCTAGTCCGTTATCAACTTGAAAAAGTGGCACCG
AGTCGGTGCTTTTTTTCCCTGCAGGAGAGTAATGTACTTACCTTTGGGGATTTCATAACTAAAAGCGG
CAGAAGAACACCATTTTTTATAAATACAGGTAACTACAAGACAGGTAATCAATTAAATAAGTTGGCT
AAGTTTTATGCTAAAGCAATATATGATAATTTTGGAGATGATTTTGATATTTTATTTGGGCCTGCATAT
AAAGGAATACCTTTAAGTGTTTCAGTAGCTATGGCACTTGATAATATTTATGGAATTAATGCAGCTTA
TTGTTCAAATAGAAAAGAAGTTAAAGATCACGGTGATAAGGGAATACTTCTTGGAGCAAAGCTTGAA
GAAGGAGACAGAGTTATAATTGTAGAAGATGTCACAACAGCTGGTACATCAGTATACGAAACAATG
CCTATACTTAAATCACAGGCTGAGGTTGATGTAAAGGGAATCATAATATCAGTGGATAGAATGGAAA
GAGGTAAGGGAGATAAGAGTGCCTTAACTGAACTTAAAGAAAAGTTTGGATTTAAAACATGTTCTAT
TGTTACTATGGAAGAGGTAGTAGAATATTTGTATAAGAAAAATATCAATGGCAAAGTAATCATAGAT
GATAAAATGAAAGATAGAATTAATGAGTACTATAAAGAGTATGGAGTAAAATAGTAAGCGGCCAGG
GAATTCCCGGGTCGACGTGTAAGCTCCTGCAGACTAGTTCACACTGGCTCACCTTCGGGTGGGCCTTT
CTGCGTTTATATACTAGAGAGAGAATATAAAAAGCCATTAAGATTATATTTCATTAATTAAAGTTAAA
TTTTGTATATTCAAAAAGCTCTTGATATTTACGGCAGAGCCACCATATAGAGATGCCTCAGACCCTAA
TGATGAAATTTCTATATTTATTCCTTTGTTAAAAGAGCTAACTAACATATCTTTTGTAATCTGTAATATC
TCAGGGATATCACTAATTATTTGGCTATTTAAATATATTATTTCAGGAGCATAAGTTGTAATAGCATTA
TTTATAGCTATTGTTAAGTAACTACAAAACTCATGAATAACTTTTTTGGCATTCTGGTTATCTTCATAAT
ATAGTTGCTTTACTATATCAGAATCTATTTTAGGAATATTTTCTAAAGAAGAAAGCTGTTCAAATACCT
TTTTTTCTGAACAATACTGTTCTAAACAGCCACGATTTCCACAAGGACATAGTTTACCATTAGGCATG
ATGATAGTGTGACCAATTTCTCCACTCATACCATTTCTGCCACTATATAATTTATTATTTATTATAATGC
CTGAACCAAATCCACTGTGAATACTGAGACTAAGTAGTGAGTTATGTACAGTTGAAAAAGTATTCTC
AGCTAAAGCTGTTAAGTTTGCTTCATTTTCTATATGAATAGGAAAGTCATACTTTTTGCTAAGGATGCT
ATATAAGTCAATCTCATTAAGATTGTAATATGGAGTAAACAATACTTTATTTTCACAAGTAATCCCAT
GTATAGCCAAAGTAAGACCAATTACCTTATAAGGAGTGTCTATTTTGGATATGTTATAACTATTTATA
ATTTCATCTATCAATTGTATAACATTGTCTTTACTTACTTGTATATCTGTTAATTTCTTAGAATTTATAAT
AGTTCCATCTAAGTAAGATAGAGAAGAAAATATATAGTCGTATCCAACGTCCATACTTAAAGAAATC
CCTGCACATTTATTAAATACTAATAATATGGGTTTTCTTCCGCCACTATGAGTACTATTTCCAATTCCT
ATTTCATGAACTAGAGATTCATCAATAAGTTTTTTGGTAATAGCGGATATAGTTGCTTTATTCAATCCT
ATAGTAGAAGCTATACTTGCCCTAGAAATAGGACCATTTTTTATAATTTGTTCAAGTACCAATCTTTCA
TTCATTTCTCGAATAGTGTATTTATCAGTAACCAATTTGATATTCCTCCTTAAAATAATATTGTAATACT
TTTTACACAAAAATAAAAGGTTATTTTGCATTGACAAAGATAATTAAATATTTTATTATTAGTTCATAA
GTTAGTTTAATATACTAACAAAAATAAAGCAAGTAAAATATACCTAAAATATAAAAAAATTAGGATA
GGAAAACGATAGTTATGAAGTGGCATTCAAGGAGGGATGAAGTATGGATAAGAAATACTCAATAGG
CTTAGATATCGGCACAAATAGCGTCGGATGGGCGGTGATCACTGATGAATATAAGGTTCCGTCTAAA
AAGTTCAAGGTTCTGGGAAATACAGACCGCCACAGTATCAAAAAAAATCTTATAGGGGCTCTTTTAT
TTGACAGTGGAGAGACAGCGGAAGCGACTCGTCTCAAACGGACAGCTCGTAGAAGGTATACACGTC
GGAAGAATCGTATTTGTTATCTACAGGAGATTTTTTCAAATGAGATGGCGAAAGTAGATGATAGTTTC
TTTCATCGACTTGAAGAGTCTTTTTTGGTGGAAGAAGACAAGAAGCATGAACGTCATCCTATTTTTGG
AAATATAGTAGATGAAGTTGCTTATCATGAGAAATATCCAACTATCTATCATCTGCGAAAAAAATTG
GTAGATTCTACTGATAAAGCGGATTTGCGCTTAATCTATTTGGCCTTAGCGCATATGATTAAGTTTCGT

GGTCATTTTTTGATTGAGGGAGATTTAAATCCTGATAATAGTGATGTGGACAAACTATTTATCCAGTTG
GTACAAACCTACAATCAATTATTTGAAGAAAACCCTATTAACGCAAGTGGAGTAGATGCTAAAGCGA
TTCTTTCTGCACGATTGAGTAAATCAAGACGATTAGAAAATCTCATTGCTCAGCTCCCCGGTGAGAAG
AAAAATGGCTTATTTGGGAATCTCATTGCTTTGTCATTGGGTTTGACCCCTAATTTTAAATCAAATTTT
GATTTGGCAGAAGATGCTAAATTACAGCTTTCAAAAGATACTTACGATGATGATTTAGATAATTTATT
GGCGCAAATTGGAGATCAATATGCTGATTTGTTTTTGGCAGCTAAGAATTTATCAGATGCTATTTTACT
TTCAGATATCCTAAGAGTAAATACTGAAATAACTAAGGCTCCCCTATCAGCTTCAATGATTAAACGCT
ACGATGAACATCATCAAGACTTGACTCTTTTAAAAGCTTTAGTTCGACAACAACTTCCAGAAAAGTAT
AAAGAAATCTTTTTTGATCAATCAAAAAACGGATATGCAGGTTATATTGATGGGGGAGCTAGCCAAG
AAGAATTTTATAAATTTATCAAACCAATTTTAGAAAAAATGGATGGTACTGAGGAATTATTGGTGAA
ACTAAATCGTGAAGATTTGCTGCGCAAGCAACGGACCTTTGACAACGGCTCTATTCCCCATCAAATTC
ACTTGGGTGAGCTGCATGCTATTTTGAGAAGACAAGAAGACTTTTATCCATTTTTAAAAGACAATCGT
GAGAAGATTGAAAAAATCTTGACTTTTCGAATTCCTTATTATGTTGGTCCATTGGCGCGTGGCAATAG
TCGTTTTGCATGGATGACTCGGAAGTCTGAAGAAACAATTACCCCATGGAATTTTGAAGAAGTTGTCG
ATAAAGGTGCTTCAGCTCAATCATTTATTGAACGCATGACAAACTTTGATAAAAATCTTCCAAATGAA
AAAGTACTACCAAAACATAGTTTGCTTTATGAGTATTTTACGGTTTATAACGAATTGACAAAGGTCAA
ATATGTTACTGAAGGAATGCGAAAACCAGCATTTCTTTCAGGTGAACAGAAGAAAGCCATTGTTGAT
TTACTCTTCAAAACAAATCGAAAAGTAACCGTTAAGCAATTAAAAGAAGATTATTTCAAAAAAATAG
AATGTTTTGATAGTGTTGAAATTTCAGGAGTTGAAGATAGATTTAATGCTTCATTAGGTACCTACCATG
ATTTGCTAAAAATTATTAAAGATAAAGATTTTTTGGATAATGAAGAAAATGAAGATATCTTAGAGGA
TATTGTTTTAACATTGACCTTATTTGAAGATAGGGAGATGATTGAGGAAAGACTTAAAACATATGCTC
ACCTCTTTGATGATAAGGTGATGAAACAGCTTAAACGTCGCCGTTATACTGGTTGGGGACGTTTGTCT
CGAAAATTGATTAATGGTATTAGGGATAAGCAATCTGGCAAAACAATATTAGATTTTTTGAAATCAG
ATGGTTTTGCCAATCGCAATTTTATGCAGCTGATCCATGATGATAGTTTGACATTTAAAGAAGACATT
CAAAAAGCACAAGTGTCTGGACAAGGCGATAGTTTACATGAACATATTGCAAATTTAGCTGGTAGCC
CTGCTATTAAAAAAGGTATTTTACAGACTGTAAAAGTTGTTGATGAATTGGTCAAAGTAATGGGGCG
GCATAAGCCAGAAAATATCGTTATTGAAATGGCACGTGAAAATCAGACAACTCAAAAGGGCCAGAA
AAATTCGCGAGAGCGTATGAAACGAATCGAAGAAGGTATCAAAGAATTAGGAAGTCAGATTCTTAA
AGAGCATCCTGTTGAAAATACTCAATTGCAAAATGAAAAGCTCTATCTCTATTATCTCCAAAATGGAA
GAGACATGTATGTGGACCAAGAATTAGATATTAATCGTTTAAGTGATTATGATGTCGATCACATTGTT
CCACAAAGTTTCCTTAAAGACGATTCAATAGACAATAAGGTCTTAACGCGTTCTGATAAAAATCGTG
GTAAATCGGATAACGTTCCAAGTGAAGAAGTAGTCAAAAAGATGAAAAACTATTGGAGACAACTTC
TAAACGCCAAGTTAATCACTCAACGTAAGTTTGATAATTTAACGAAAGCTGAACGTGGAGGTTTGAG
TGAACTTGATAAAGCTGGTTTTATCAAACGCCAATTGGTTGAAACTCGCCAAATCACTAAGCATGTGG
CACAAATTTTGGATAGTCGCATGAATACTAAATACGATGAAAATGATAAACTTATTCGAGAGGTTAA
AGTGATTACCTTAAAATCTAAATTAGTTTCTGACTTCCGAAAAGATTTCCAATTCTATAAAGTACGTG
AGATTAACAATTACCATCATGCCCATGATGCGTATCTAAATGCCGTCGTTGGAACTGCTTTGATTAAG
AAATATCCAAAACTTGAATCGGAGTTTGTCTATGGTGATTATAAAGTTTATGATGTTCGTAAAATGAT
TGCTAAGTCTGAGCAAGAAATAGGCAAAGCAACCGCAAAATATTTCTTTTACTCTAATATCATGAACT
TCTTCAAAACAGAAATTACACTTGCAAATGGAGAGATTCGCAAACGCCCTCTAATCGAAACTAATGG
GGAAACTGGAGAAATTGTCTGGGATAAAGGGCGAGATTTTGCCACAGTGCGCAAAGTATTGTCCATG
CCCCAAGTCAATATTGTCAAGAAAACAGAAGTACAGACAGGCGGATTCTCCAAGGAGTCAATTTTAC

CAAAAAGAAATTCGGACAAGCTTATTGCTCGTAAAAAAGACTGGGATCCAAAAAAATATGGTGGTTT
TGATAGTCCAACGGTAGCTTATTCAGTCCTAGTGGTTGCTAAGGTGGAAAAAGGGAAATCGAAGAAG
TTAAAATCCGTTAAAGAGTTACTAGGGATCACAATTATGGAAAGAAGTTCCTTTGAAAAAAATCCGA
TTGACTTTTTAGAAGCTAAAGGATATAAGGAAGTTAAAAAAGACTTAATCATTAAACTACCTAAATA
TAGTCTTTTTGAGTTAGAAAACGGTCGTAAACGGATGCTGGCTAGTGCCGGAGAATTACAAAAAGGA
AATGAGCTGGCTCTGCCAAGCAAATATGTGAATTTTTTATATTTAGCTAGTCATTATGAAAAGTTGAA
GGGTAGTCCAGAAGATAACGAACAAAAACAATTGTTTGTGGAGCAGCATAAGCATTATTTAGATGAG
ATTATTGAGCAAATCAGTGAATTTTCTAAGCGTGTTATTTTAGCAGATGCCAATTTAGATAAAGTTCTT
AGTGCATATAACAAACATAGAGACAAACCAATACGTGAACAAGCAGAAAATATTATTCATTTATTTA
CGTTGACGAATCTTGGAGCTCCCGCTGCTTTTAAATATTTTGATACAACAATTGATCGTAAACGATAT
ACGTCTACAAAAGAAGTTTTAGATGCCACTCTTATCCATCAATCCATCACTGGTCTTTATGAAACACG
CATTGATTTGAGTCAGCTAGGAGGTGACTGACCATGGTTGCTAGCTTCGATCGTTGAGCTCTTTCTAGA
TTCTCGAGTTGGTACCTTGGCGCCACTTAATGATTTGCCAGTAAAAGAGATTGTTTCTAGCTCTCACAT
TCTTGCAGATATAATATTGCCTAGAGCTGAAGTTATATATGATTATCTTAAGTAATAAAAATAAGAGT
TACCTTAAATGGTAACTCTTATTTTTTTAAATGTATATTGATAAAAATAATAATAGTGGGTGTAATTAA
GTTGTTAGGAGGTTAGTGGGCCCCCTTTATTCATGTTTTGAAACATTTTTATCTTTTGTGTATTTTACGT
GTAGTAATTTGTGAGCAAGTCCTTCACCTGGTTTTCCAAAGTAGCTATCATACATTTTAATAATAGCTG
GATTATCATGTGACTTTCTCTTTGAAAGAACATTTTTATCTTGGTTGTATAATACTGATGCTCTTAGTTT
TCTGTAATCAACATTTTCTCTATCAAGAGCATTTACGTGAGGTTGACCTCCACCATTTATACATCCACC
AGGGCAAGCCATTACTTCTATAAAGTGATATTGTTTTTCGTTCATTTTTCCAGATTTCATAAACTCGAA
GAAGTTAGAAGCACCATTTATAACAGCAACGTTTAGTTTATTTCCAGCAATTTCAACTTCCGCTTCTTT
TATGCCTTTAAAGCCTCTTACTTCAGTGTAATCAACATTTTCAAGTTCTTTATTTTCAGCAAAGTCTTTA
GCTGATCTTATTGCAGCTTCCATAACGCCACCGGTTGCACCAAAGATAGCTCCAGCACCACTGTAAGT
ACCCATAGCAGGATCAACTTCACCATCTTCAAGATCTGCAAATTTAATTTTTGCATCTTTAATCATTTT
TGCAAGCTCTCTTGTAGTTAAGGATGCATCAATATCTCTTAAGCTGTTAGTTTCCATGAAAGGAATATC
TGCTTCATATTTTTTATCATTACAAGGCATGATAGTAACTGTATAAACATCTTCTGGAGCTATTCCTGA
AATTGAAGGATAGTAAGTTTTTGATGCAGTACCAAATATTTGTTGTGGTGATTTTGCTGATGAAAGATT
ATCTAATAATTCAGGATGATAATTTTGAGCTAATCTTACCCATGCAGGACAGCAAGATGTAAACATA
GGGAATGGGCCATTATTTTTAACTCTGCCTAAAAGTTCAGTAGCTTCTTCCATTATAGTCATATCTGCA
CCAAAGTTTATATCAAATACTTTATCAAAGCCTAACATTCTAAGTGCAGTATATAGTTTTCCTGTTACA
TCTTTTCCATATCCCATTTTGAATAATTCGCCCATAGCAGTTCTTACTGATGGAGCCATTGCAACAATG
ACATGTTTTTTAGGGTCATTAAGAGCTTCTTGAACTTTTTCTATATGGGATTTTTCTTTTAAAGCAGCAA
CAGAAGAGC
